# Personal Health Record implementation in rural primary care: A descriptive exploratory study using RE-AIM framework

**DOI:** 10.1371/journal.pdig.0000537

**Published:** 2024-06-26

**Authors:** Selena Davis, Mindy A. Smith, Lindsay Burton, Kathy L. Rush

**Affiliations:** 1 Department of Family Practice, The University of British Columbia, Vancouver, Canada; 2 Patient Voices Network, Vancouver, Canada; 3 Department of Family Medicine, Michigan State University, East Lansing, Michigan, United States of America; 4 School of Nursing, The University of British Columbia-Okanagan, Kelowna, Canada; Taipei Medical University, TAIWAN

## Abstract

Demand is emerging for personal health records (PHRs), a patient-centric digital tool for engaging in shared decision-making and healthcare data management. This study uses a RE-AIM framework to explore rural patients and providers’ perceptions prior to and following implementation of a PHR. Health care providers and their patients were recruited from early-adopter patient medical home clinics and a local patient advisory group. Focus groups were used to explore patient and provider pre-implementation perceptions of PHRs and post-implementation provider perspectives. Patients were invited through participating clinics to use the PHR. An implementation process evaluation was conducted. Multiple methods and data sources were used and included pre-/post-intervention patient surveys, provider interviews, and PHR/EHR administrative data. Both patient and provider focus groups described PHRs as providing a comprehensive health story and enhanced communication. Patients prioritized collection of health promotion data while providers endorsed health-related, clinical data. Both groups expressed the need for managing expectations and setting boundaries on PHR use. The evaluation indicated *Reach*: 16% of targeted patients participated and an additional 127 patients used the PHR as a tool during the COVID-19 pandemic. *Effectiveness*: Patient satisfaction with use was neutral, with no significant changes to quality of life, self-efficacy, or patients’ activation. *Adoption*: 44% of eligible clinics participated, primarily those operated publicly versus privately, in smaller communities, and farther from a regional hospital. *Implementation*: Despite system interoperability expectations, at time of roll out, information exchange standards had not been reached. Additional implementation complications arose from the onset of the pandemic. One clinic on-boarded additional patients resulting in a rapid spike in PHR use. *Maintenance*: All clinics discontinued PHR within the study period, citing several key barriers to use. RE-AIM offers a valuable process evaluation framework for a comprehensive depiction of impact, and how to drive future success. Interoperability, patient agency and control, and provider training and support are critical obstacles to overcome in PHR implementation.

## Author summary

In phase one, our research study identified the similarities and differences between patients and primary care providers in Personal Health Record technology use and functionality to guide implementation. In phase two, we used an implementation science framework benefitting from multiple methods, data types, and an extensive set of data points, to conduct an implementation process evaluation. The process evaluation of a Personal Health Record implementation offered a comprehensive depiction of its impact and how and in what ways the intervention worked and didn’t. The evaluation described the characteristics of individuals willing to participate (Reach), its success in achieving intended outcomes (Effectiveness), the number and representativeness of settings and individuals initiating use (Adoption), the extent that the intervention was delivered as intended (Implementation), and whether there was continued use of the system beyond the study (Maintenance). Overall, our study enhances understanding of PHR implementation strategies within rural primary care with opportunities of future refinement of PHR implementations and improved implementation outcomes.

## Background

There is an increasing demand for digital health tools that enable patients to optimally participate in decision-making and management of their healthcare with their care team. A personal health record (PHR) is a patient-centric digital health technology that supports this vision [1]. PHRs are architected as standalone, tethered (attached to a provider’s electronic health record (EHR), or interconnected (integrated with multiple health information systems, including EHR). The PHR, if optimally designed and used, enables the patient to access, securely communicate with care providers, self-manage, and contribute to their health record [2]. Although outcome data are limited, PHR use has been shown to decrease emergency room visits and preventable hospital stays in patients with chronic diseases [3], and improve patient satisfaction and disease control for conditions such as diabetes, asthma, and hypertension [4]. Little is known about use of PHRs in primary care settings in Canada, specifically in British Columbia’s (BC) newly realized patient-centred, team-based primary care model [5].

Geographic rurality heightens the need for digital enablement of primary care services. Factors such as more chronic disease multimorbidity and obstacles to receiving healthcare (e.g., limited access to health services and provider shortages) are well-known in rural communities relative to their urban counterparts [6,7]. Health information technologies, like PHRs, are seen as an important component for addressing these health disparities [8]. However, despite the benefits for improved health and access, rural residents appear less likely to electronically communicate with doctors and manage personal health information online compared to urban residents [9]. Cited reasons include lack of broadband access in rural areas and lower income and education among rural residents compared to their urban counterparts [10]. In addition, several barriers to PHR use have been identified including lack of rural provider recommendation [11], despite similar rates of maintaining an electronic medical record system [12]; lack of meaningful pre-implementation involvement and decision making; and lack of engagement strategies during and after deployment [1,13]. Finally, provider age and institutional affiliation may be additional factors. Rural physicians are older and more likely to be in solo practice with limited options for reimbursement for time spent on the computer or for staff support [14], factors important in adoption of PHRs [15].

Since the COVID-19 pandemic, uptake of virtual care has skyrocketed, although most often conducted using telephone over video visits to overcome broadband access issues in rural communities [16]. Even with a shift in type of care visit, patient-facing digital solutions for improving healthcare remain relatively unexplored in Canada. The impetus for this study was an opportunity to enhance patient engagement within the transformation and digital enablement of team-based primary care in rural BC communities. To assess the application, uptake, and impact of a PHR intervention on patients and providers, we needed a robust process evaluation framework to ensure that all aspects of the implementation were evaluated [17]. One widely used and validated framework is RE-AIM (an acronym for the dimensions of Reach, Effectiveness, Adoption, Implementation, and Maintenance) [18]. *Reach* focuses on the characteristics of individuals willing to participate in the intervention, *Effectiveness* is an assessment of how well the intervention achieves intended outcomes, *Adoption* assesses the number and representativeness of settings and individuals willing to initiate an intervention, *Implementation* focuses on the extent that the intervention is delivered as intended, and *Maintenance* measures the extent to which an intervention is sustained over time. Although prior studies on adoption of PHRs have examined numerous personal, technological, and organizational factors related to objectively measured PHR uptake, authors of a systematic review were only able to draw definitive conclusions about the influence of 12/105 factors and found a lack of use of theoretical frameworks and attention to continued use [19]. RE-AIM offers a structure in which to capture a comprehensive depiction of the impact of a PHR implementation [20], and enables evaluators to determine how and why an intervention works, permitting future refinement [21].

The overall aim of our two-phased, multi-methods exploratory research was to identify priorities for PHR functionality and implement a PHR in clinical practice to gain insights into the meaning, value, and use of a PHR and patient-generated data for patients and providers. To that end, this paper offers a comparison of patient and provider pre-implementation perspectives of PHRs identified in the first phase of our research and reports on the implementation of a PHR in a rural team-based primary care setting from the second phase of study. The study objectives were to: (i) describe similarities and differences in PHR perceptions of patients and providers before implementation, (ii) describe the PHR implementation, and (iii) explain its impacts including perceived benefits and barriers, patient engagement, satisfaction with use, and types of use. The preferred functions of PHRs from the provider perspective, identified in phase 1, have been reported separately [22].

## Methods

### Setting

Phase 1 of the study was conducted in November 2019, pre- COVID-19, in five rural communities of the interior health region of BC. The phase 2 implementation and evaluation took place in four team-based primary care clinics in this same region, between Jan 2020-Oct 2020. The multi-provider clinics serve as patient medical homes (PMH) [5]—family practices that provide team-based, patient-centred, comprehensive, longitudinal care, as their care model. Each clinic uses a separate EHR system with very limited interoperability between any two clinics or between a clinic and the hospital clinical information system. Harmonized ethics approval was granted by the joint review boards of University of British Columbia Clinical Research Ethics Boards (H19–00958) and Interior Health Research Ethics Board.

### Study design

**Fig 1:**
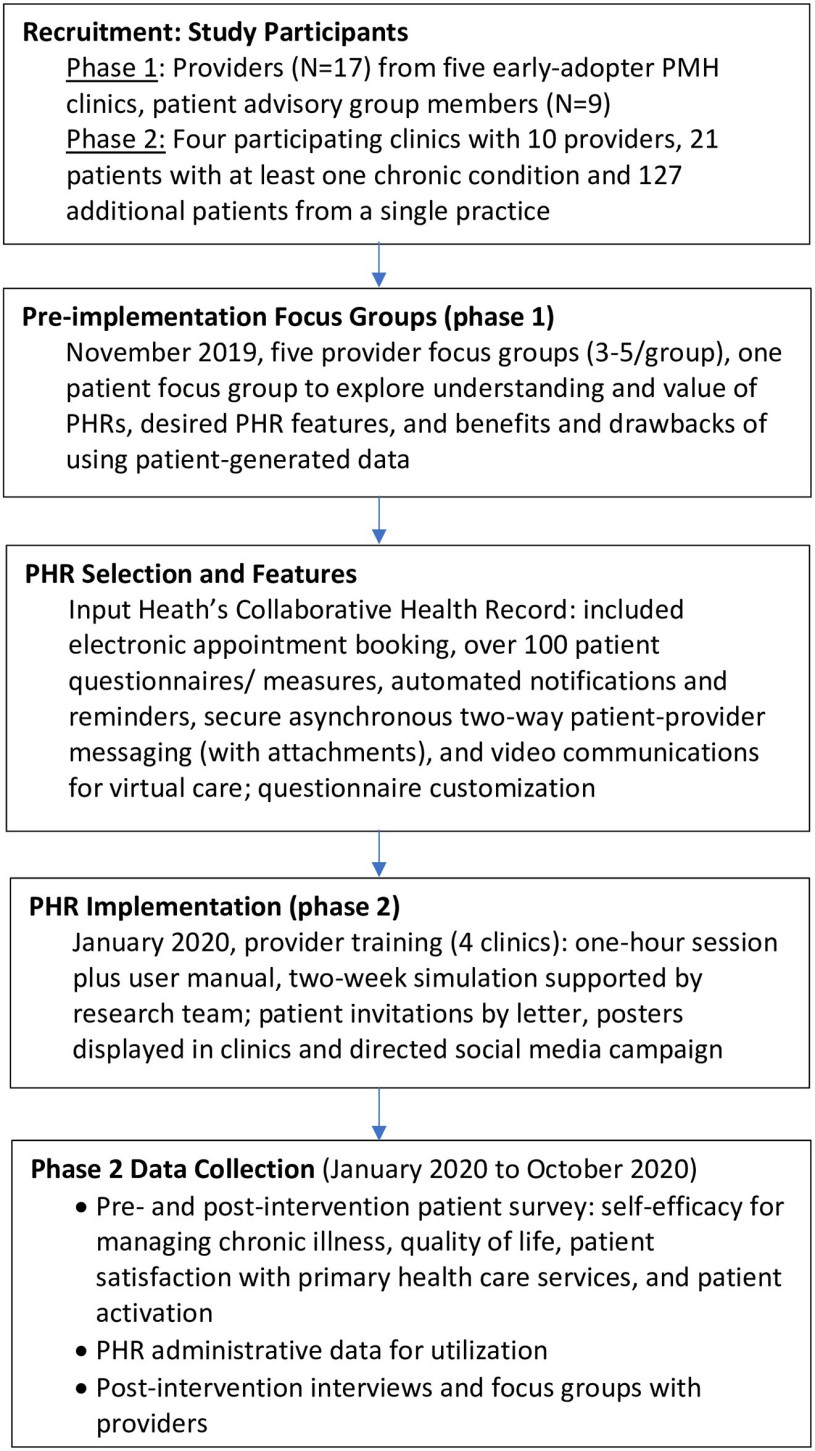
Study Design Summary.

A qualitative descriptive design via focus groups was used in phase 1 to capture and compare rural patient and provider perceptions of PHRs prior to implementation. A PHR was then integrated into participating clinics and its implementation and impact examined (phase 2) using multiple methods and data sources (Fig 1 and Table 1). Notably, phase 2 of the study took place during the COVID-19 pandemic which resulted in a significant increase in virtual healthcare.

To structure the process evaluation of phase 2, we applied the RE-AIM framework to capture a comprehensive picture of a PHR implementation and its impact in rural clinical practice. To be maximally useful, it is important for all aspects of the 5-dimensional framework to be incorporated into the planning and analysis of the process evaluation [23]. This mixed methods evaluation set out to describe all 5 dimensions with multiple criteria within each dimension.

**Table 1:** Evaluation dimensions, measures, and data sources of the RE-AIM framework.

RE-AIM Dimension	Level	Description	Data collection timeframe	Data Source	Data collected
**Reach**	Patient/ individual	Characteristics of patients invited who participated	T0 –pre-intervention	Patient Pre-survey	1. Health history 2. Demographics 3. PHR familiarity and use expectations
	Patient/ individual	Percentage of patients invited who participated	T1 –during	PHR/ EHR Administrative data	4. No. of patients with one or more chronic conditions identified from EHR problem list 5. No. of invited patients who used PHR
	Patient/ individual	Description of patients who participated as a result of provider request due to COVID-19	T1 –during	PHR/ EHR Administrative data	6. No. of patients who used PHR 7. Demographics
**Effectiveness**	Patient individual	Self-report measures by patients who were invited (target population) and participated	T0 –pre-intervention T2 –post-intervention	Pre-, Post-Patient survey	8. Self-efficacy 9. Patient Activation 10. Quality of Life 11. Satisfaction with primary health care services
		Description of PHR use by all participants	T1 –during	PHR Administrative data	12. Types of patient-generated data and PHR features used
		Perception of PHR value & use by target population	T2 –post-intervention	Post-Patient survey	13. Satisfaction with PHR use 14. Satisfaction with PHR features 15. Value of PHR
	Provider individual	Perception of benefits, barriers, and use by providers	T2 –post-intervention	Interview/ Focus Groups Email/phone correspondence	16. PHR use, benefits, and barriers
**Adoption**	Provider/ individual	Characteristics of providers invited who participated	T0 –pre-intervention	Division of Family Practice stats	17. Provider type 18. Biological Sex 19. Experience with technology 20. Time in clinical practice 21. Time using EHR
		Percentage of providers invited who participated	T0 –pre-intervention	Division of Family Practice stats	22. Number of providers who were invited and participated
	Setting/ organizational	Percentage of PMH Clinics invited to participate	T0 –pre -intervention	Division of Family Practice stats	23. Total number of PMH clinics in rural health region
		Characteristics of PMH clinics invited to participate (compared to those that decline participation)	T0 –pre-intervention	Division of Family Practice stats	24. Clinic location (rurality) 25. Length of time as PMH 26. Clinic ownership 27. Physician full-time equivalent (FTE)
		Description of issues arising with acceptance of intervention by clinic	T1 –during	Division of Family Practice stats Administrative Data	28. Adoption issues related to system interoperability and COVID-19 pandemic
**Implementation**	Setting/ organizational	Description of the implementation	T1 –during	PHR Administrative Data	29. No. patients given initial access by month 30. Implementation timeframe, training, and system adaptations
		Implementation impact to RE-AIM dimensions of Adoption and Maintenance	T1 –during	Division of Family Practice stats PHR Administrative Data	31. Implementation fidelity and issues arising due COVID-19 pandemic
**Maintenance**	Setting/ organizational	Percentage of clinics interested in continuing to use	T2 –post-intervention	Interview/ Focus Groups Email/ phone correspondence	32. Continued PHR use

#### Personal Health Record intervention used in phase 2 implementation

Currently, there are no commercially available, interoperable PHRs in BC and the provincial health information exchange standards are in their infancy. The existing EHRs of participating clinics did not integrate with any PHRs or patient portals. The research team selected a PHR, Input Heath’s Collaborative Health Record [24], which has since been acquired by TELUS Health, that had a collaborative and patient-centred perspective and an interest in advancing PHRs, architected within an interconnected digital health ecosystem. For this study, the selected PHR remained architected as a stand-alone system–i.e., it was not interoperable with the clinic’s EHR system. Every effort was made to consider desired features, utility, and identified benefits uncovered in phase 1 for implementation.

The PHR functionalities are listed in Fig 1. Providers were given the opportunity to tailor questionnaires and forms–e.g., patient pre-visit form or patient consent form permitting the sharing of electronic patient-generated data between patient and provider. At various times during the study, providers sent messages to patients via the PHR to complete questionnaires or measures (e.g., blood pressure, General Anxiety Disorder-7) or submit an image.

#### PHR implementation schedule & training

Implementation began with provider training in use of the PHR (Fig 1). Training consisted of an orientation (e.g., how to enroll a patient, how to send messages) and simulations with test patients. For a minimum of two weeks following the training session, providers were able to use the PHR through simulation supported by a research team member. Patient enrollment began immediately afterwards. For three months following training, a research team member had monthly check-ins with providers to troubleshoot and answer questions.

Patient access and PHR support was managed by the clinic once patient consent to participate in the study was received by the research team. Patients used their login information to access the PHR and they were provided with basic instructions from clinic staff on using the PHR. Patients and providers were directed to contact the research team with any questions

### Recruitment & Participants

For both phases, a pragmatic approach was used to recruit rural, community team-based clinics. These multi-provider clinics typically comprise physicians, nurse practitioners, registered nurses, and allied health professionals. The rural interior Kootenay Boundary Division of Family Practice [25] invited their nine early-adopter clinics of a PMH model of care to participate. These clinics had a particular interest in digitally enabling their clinical practice and the capacity to take on the project requirements.

In phase 1, all providers (physicians, nurse practitioners, social workers, physiotherapists, mental health professionals, registered nurses) in the nine early-adopter PMH clinics were invited by email to participate in provider focus groups. All members of a Patient Advisory Committee to the Kootenay Boundary Collaborative Services Committee, composed of patients living in this region, were invited to participate in a patient focus group.

In phase 2, patients with one or more chronic conditions (e.g., hypertension, chronic obstructive pulmonary disease, heart failure, diabetes) identified from the EHR problem list of the four participating PMH clinics were targeted for recruitment. Chronic disease was identified as a focus for the Kootenay Boundary Division of Family Practice and patients with these conditions were identified as typically having more frequent clinic visits and therefore were more likely to offer a broad look at patient experiences and PHR engagement. Patients of participating clinics were invited via letters sent by the clinic using common communication methods and posters displayed at clinics, as well as a directed social media campaign (https://youtu.be/_PgIbovINe0).

Notably, one month into the study, one of the participating clinics requested use of the PHR to support the significant shift to virtual care at the beginning of the COVID-19 pandemic. As such, providers also took to convenience sampling, by inviting individual patients presenting for virtual care as the pandemic took hold. These additional patients were not assessed for a chronic condition but were included in the study.

### Data collection

In phase 1, following written informed consent, one patient focus group and five primary care provider focus groups were held at mutually convenient times. Basic demographic data were collected from all patients (e.g. sex, health status, technology use) and providers (e.g. years in clinical practice, technology user type). Focus groups were audio-recorded and lasted approximately 1 to 1.5-hours each. Focus groups were facilitated by the research team using a semi-structured interview guide (S1 Appendix). PHRs were defined broadly, in the participant consent form and introduction letter, as a digital tool for patients to capture their heath data, and where they have access to enter information as well as manage appointments, communicate with providers, and view test results. After initial discussion, functions of a PHR under consideration for implementation in phase 2 were demonstrated, and participants were asked additional questions around features and utility and to aid in tailoring functionality.

In phase 2 and to address each RE-AIM dimension for the PHR implementation, we drew on multiple date collection methods and data sources. The structure for our data collection is summarized in Fig 1 and measures are listed in Table 1. Patient survey measures are listed in Table 1 and described in S2 Appendix. The pre-intervention survey also captured basic demographics and health history, along with 15 questions about PHR familiarity and expectations using Likert scales—e.g., “A PHR will improve communications with my primary care provider” and “How often do you expect to use the PHR?”. The post-intervention survey also captured PHR usage satisfactions via 18 questions using Likert scales–e.g., “My primary care provider responded to data that I entered in the PHR” and “Compared to what I expected, I used the PHR more or less than expected”. The interview guide used to capture qualitative data from post-intervention interviews and focus groups with providers is shown in S3 Appendix.

No pre- or post-intervention patient survey data were collected from the additional participants invited by providers at one clinic due to COVID-19, but the PHR administrative data were gathered (i.e., deidentified age, gender). These additional participants’ usage data were included within ‘total population’ PHR usage for the study. The PHR vendor provided the PHR administrative data for all participants.

### Data analysis

In phase 1, interview data were transcribed, and open coding of the transcripts within NVivo Version 12 was completed for units of meaning (e.g., words, phrases). The initial codes were compared and clustered into themes and subthemes by one research team member (LB) to develop a preliminary coding framework. Iterative refinements were made using consensus among research team to the themes and subthemes as coding progressed to account for all participant data.

The multiple data sources in phase 2 were analyzed according to their data type and the results presented by dimension of the RE-AIM framework. The pre- and post-intervention patient survey of the target population, including demographic and use data, were analysed by one research team member (LB) using R for simple descriptive statistics (e.g., means, frequencies, percentages).

Inductive thematic analysis of the qualitative data, inclusive of provider focus groups and individual interviews, and email correspondence, was used to guide the interpretation and representation of textual data [26]. Interviews were audio recorded and transcribed. Themes emerged from the inductive analysis of the data according to the developed coding frame and repeating ideas. Quotations of the participants that best illustrated the themes were used. The coding scheme included the concept codes of effectiveness of use, benefits, and barriers. Representative quotations from the participants for the resulting topics were identified

## Results

### Phase 1: Pre-implementation

The patient focus group (n = 9) was 89% female and had a median age of 64 years. Most were college educated although income varied widely, overall health ratings were evenly split across fair, good, and excellent, and evenly split on use of technology, either always or regular (S4 Appendix). Across five provider focus groups representing five clinics, providers (n = 17) were predominantly female (82%), from different professional categories, with a median duration of practice of 9.5 years, and 53% identifying themselves as an average technology user (S4 Appendix). Patients and providers shared similar and differing views and understandings of PHRs based, in part, on their different contexts, exposures, and experiences with PHR features (Table 2). Two themes were constructed to represent patients’ and providers’ views of PHRs: (i) patient-centric focus and control, and (ii) the value of communication and information sharing with boundaries.

**Table 2:** Views of Personal Health Records by Patients and Providers.

Patients	Providers
Similar Views: Housing a Comprehensive Patient History	
“Ultimately … your total health should be there because … If there’s missing pieces, then it can all fall apart … so it needs to be comprehensive” (Patient 2, 82 yrs. male) “Repeating the same thing over and over again to different people is, like, triggering for a lot of things; you could potentially go forward way faster” (Patient 1, 36 yrs. female)	“Instead of waiting for a consult note …to be able to, in real-time, access what’s going on …it would be a great place to have all that information” (Provider 3, 22 yrs of practice)
Similar Views: Flexible Communication	
*“*… the email back and forth to the health providers, I’ve done that, and that’s, I think, essential” (Patient 4, 55 yrs. female) “Technology is helping bridge communication and get everyone out of the dark ages” (Patient 1, 36 yrs. female)	“A lot of people … say, ‘if you get worse, go to Emergency and if you’re doing okay, just see me in a week’ … Something like that when you’re not really sure which way it’s going to go and then you don’t hear from them … It’s going to make you feel better if you could be, like, ‘Hey. Where are you?’” (Provider 1, 9 yrs of practice)
Differing Views: Types of Patient-Generated Data Desired	
Health promotion data “I think the information that I keep is more around a health promotion, wellness perspective, whereas if I compared that to what’s in the health record currently, it would all be around sickness and illness” (Patient 8, 66 yrs. female)	Clinical data Pre-visit clinical information gathering, emergency department visit tracking or disease-specific questionnaires
Differing Views: Agency and Control	
Patient should control and own PHR data and be able to make changes “You should be able to sit and be the gatekeeper and just say, ‘Here’s the access to my file, you know, go for it.’ … that would transfer the information without having to go through some kind of authority. If it’s your file, you should be able to do that.” (Patient 2, 82 yrs. male)	Range of opinions: read-only, information input and sharing, PHR ownership “That’s my Nirvana dream… [patient] input, make changes, edit, access information, share that information with other providers” (Provider 10, 16 yrs of practice) “Every patient has ownership over their health records… But the issue is…does the person actually have access to them whenever they want, whereas [now], we’re a gatekeeper … they’re allowed to have anything they want, but they have to come, we’ve got to photocopy it or put it on a [USB-] stick for them.” (Provider 5, 10 yrs of practice)
Differing Views: Boundaries and Limit Setting	
Limit setting “to wall off areas, if you will that we just share with our mental health provider or areas that we want to make open to others, but it should be under the patient’s control as to who gets what.” (Patient 4, 55 yrs. female)	Workflow disruption and added time “We were quite worried about what they would be messaging back. We thought we would be hit out there. They’re [patients] not; they’re not abusive” (Provider 13, 4 yrs of practice)
Differing Views: Concerns about Patient Impact	
Past negative relationships with providers might prevent “a fresh perspective of the patient” (Patient 9, 64 yrs. female)	“When patients have access to their records with no sort of formal education, it’s an anxiety creator… some patients who will see something in the portal and not understand what it says, and they’re seeing it before they’ve had time to discuss it with their doctor, and they’re quite worked up and need to be seen right away.” (Provider 15, 23 yrs of practice)

Both patient and provider groups described the PHR as providing a comprehensive health story with greater accuracy and currency of information. Differing yet complementary orientations to patient-reported data were evident. Health promotion data (e.g., food diaries, activity tracker) were important to patients, while providers endorsed health-related, clinical data (e.g., blood pressure, blood sugar) as their priority. Providers and patients both viewed the PHR as giving patients control, ownership, and access to their information. However, while providers acknowledged patients’ right to access this information, they conceded that they were currently the records’ gatekeepers.

Unique to providers was viewing the PHR’s potential to empower patients by encouraging their participation in care planning and decision-making. Some providers expressed concern over burdening patients with unnecessary anxiety resulting either from inability to interpret information or from misinterpretation of health information. Both groups wanted the PHR to include functions for bidirectional communication. For patients, the PHR afforded seamless communications for care continuity. Providers perceived the flexible communication of the PHR as promoting greater office efficiency and highlighted types of patients who would benefit from virtual follow-up through the PHR rather than in-person visits (i.e., those who are socially or geographically isolated, have anxiety about coming into the office, elderly patients who are uncomfortable driving). Both groups also expressed the need for managing expectations and setting boundaries on PHR use. Patients described the desire to share data with their providers but wanted to set limits on this sharing, whereas providers were concerned about workflow interruptions and the extra time required to communicate with their patients.

### Phase 2: Implementation and evaluation

The results are organized around each dimension of the RE-AIM framework.

#### Reach

One hundred and thirty-three patients identified as having one or more chronic conditions were invited to participate. Of those, 21 (16%) participated in the PHR use. The characteristics of targeted patients (n = 21) and for all participants (target population plus an additional 127 participants onboarded by participating providers due to COVID-19) are displayed in Table 3. The average age of the target population was 67 years with most of them married, women, and of Caucasian ethnicity. The target population also had an average of 2.5 chronic health conditions and travelled an average of 26 kms to their provider’s clinic. By comparison, the average age of all participants (n = 148) was 47 years with only slightly more female participants. Notably, one clinic onboarded the larger number of all additional participants to the PHR.

**Table 3:** Characteristics of Participating Patients.

	Target population ^a^ (n = 21)	All participants ^a^ (n = 148)
**Gender**		
Man	8 (38.1%)	63 (42.5%)
Woman	13 (61.9%)	85 (57.5%)
Other	-	-
**Age** ^b^	67.0 (7.5)	46.9 (21.7)
**Participant Clinic**		
Clinic 1	-	11 (7.4%)
Clinic 2	16 (76.2%)	19 (12.8%)
Clinic 3	2 (9.5%)	92 (62.2%)
Clinic 4	3 (14.3%)	26 (17.6%)
**Martial Status**		
Single (never married)	2 (9.5%)	-
Married	13 (62%)	-
Widowed	2 (9.5%)	-
Divorced	4 (19%)	-
**Race/Ethnicity**		
African-Canadian	1 (4.8%)	-
Caucasian	18 (86%)	-
Hispanic	1 (4.8%)	-
Missing	1 (4.8%)	-
**Education**		
Less than high school	1 (4.8%)	-
Completed high school	7 (33%)	-
Some college	5 (24%)	-
College/University graduate	8 (38%)	-
**Income**		
Less than $25,000	8 (38%)	-
$25,000-$50,000	11 (52%)	-
$51,000-$75,000	2 (9.5%)	-
**Distance to Clinic (kms)** ^b^	26 (46)	-
**Number of Chronic Health Problems**	2.5 (0.8)	-

^a^ n(%),

^b^ mean (SD)

Pre-PHR implementation patient participants (n = 21) had a high comfort (scale 1–7) with using computers (mean = 6.33, SD = 0.8). Only 43% of participants had heard of PHRs prior to the study, and only 19% had ever used a PHR. Most participants (86%) expected to use the PHR sometimes or often.

#### Effectiveness

We explored effectiveness through changes to patients’ quality of life; self-efficacy related to management of their chronic condition; and satisfaction with the PHR, its features, and its value. Pre-/post-measures were analyzed for 12 patients who completed both surveys.

No significant changes were observed in quality of life or in physical or mental health ratings between pre- and post- PHR implementation. Pre-implementation, participants reported that in the past 30 days they had an average of 15 “not good” mental health days in comparison with 19 “not good” mental health days post-PHR implementation. Self-efficacy was scored on a scale of 1(low) -10 (high). Post PHR implementation (mean = 8.02, SD = 1.81) self-efficacy scores were generally higher but not significantly different, t = -0.09, p = 0.92 from pre-PHR implementation scores (mean = 7.96, SD = 1.77). Patient activation scores, using a scale from 1–9, were generally high and unchanged (pre-PHR mean = 6.92, SD = 1.93 and post-PHR mean = 6.92, SD = 1.51). Patients’ satisfaction with their provider did not change, with participants rating their provider as “very good” or the “best provider possible” at both time periods. Patient satisfaction with the PHR use and features and perception of value were measured post-intervention using a 5 point-Likert anchored at both ends by strongly agree [score = 5] and strongly disagree [score = 1]). Scores for satisfaction and value were neutral overall: PHR use (M = 3.11; SD = 0.89), PHR features (M = 3.40; SD = 0.43), and PHR value (M = 3.57; SD = 0.94).

In terms of type of the PHR functionality used, patient-generated data (e.g., patient measures, questionnaires, and photos) were gathered and transmitted more often than booking a care visit or sending a message to the care provider (Table 4).

**Table 4:** PHR Functions Used.

Type of Use	PHR Use by target population ^a^ (n = 21)	PHR Use by all participants ^a^ (n = 148)
**Patient-generated Data Submitted**		
** Total number submitted**	**25 (100%)**	**173 (100%)**
Clinic 1	-	9 (5.2%)
Clinic 2	24 (96.0%)	34 (19.7%)
Clinic 3	1 (4.0%)	127 (73.4%)
Clinic 4	-	3 (1.7%)
**Virtual Visits Booked**		
** Total visits booked**	**3 (100%)**	**58 (100%)**
Clinic 1	-	12 (20.7%)
Clinic 2	-	4 (6.9%)
Clinic 3	-	2 (3.4%)
Clinic 4	3 (100%)	40 (69.0%)
**Messages Sent**		
** Total messages sent**	**5 (100%)**	**57 (100%)**
Clinic 1		15 (26.3%)
Clinic 2	4 (80.0%)	17 (29.8%)
Clinic 3	1 (20.0%)	14 (24.6%)
Clinic 4		11 (19.3%)

^a^ n(%)

Providers used (or didn’t use) the PHR very differently across the four participating clinics; reported usage ranged from not using, through used initially, to surprised how much they used it and would use it even more if it was integrated with the EHR (Table 5). Benefits were described as offering patients educational resources and an ease to communication with their care team. Providers perceived that sharing of data, like photos, may prevent an urgent care centre or emergency room visit. Reported barriers included lack of interoperability between patient and provider systems and negative effects on provider workload, workflow, and lack of financial incentivization.

**Table 5:** Provider Perceptions of Barriers, Benefits, & Use.

Clinic	Key Statement of Use & Quotation		Key Benefits of Use & Quotation		Key Barriers to Use & Quotation	
**Clinic 1**	Rare use in the beginning	“There isn’t much advantage to the video platform as it takes quite a bit of time” (Provider 4)	-	-	Time; no financial incentive; not integrated; more work [for virtual care] than Zoom video conference	"Didn’t offer enough that I would it’s not worth it to me to create a patient profile in there and to kind of go into a separate EHR" (Provider 7)
**Clinic 2**	Not using		-	-	Lack of patient interest; lack of integration; technical difficulties/challenges	"And of course, telephone use for me is quite easy. And so I reverted to what I knew and what was easy" (Provider 9)
**Clinic 3**	Using it regularly, would use more if integrated	"Surprised how much we are using it, almost every day" (Provider 5)	Providing educational resources	"Without the use of photos, those patients would have likely been directed to urgent care or the Emergency Department" (Provider 5)	More administrative work, integration is lacking	-
**Clinic 4**	Used in the beginning	-	More patient-centered; ease of connecting virtually; intuitive and pleasing design	“It does feel like a more patient-centered EHR compared to other EHRs.” (Provider 6)	Reverted to what is easiest when things got busy, i.e., telephone; lack of integration between systems; Technical difficulties with own equipment	"But then as things started getting busy again, we just gravitated to the easiest thing, which became the telephone, which I’m sure is no surprise to you" (Provider 2)

#### Adoption

Four of the nine invited PMH early-adopter clinics (44%) participated. The average number of physicians per clinic was two, however, physician full-time equivalent working hours varied by clinic with one having a full-time physician, others part-time physicians, and one clinic comprised nurse practitioners only. Ten of the 14 (71%) providers at the participating clinics took part in the study (five physicians, three nurse practitioners, two registered nurses). Most (70%) were female and their experience with technology varied from one reporting basic to three reporting advanced. The median length of time in clinical practice was 9 years and median length of time using an EHR was 7 years. The characteristics of participating and non-participating clinics are shown in Table 6.

**Table 6:** Characteristics of Participating Clinics.

Characteristic	Participated N = 4 ^a^	Declined N = 5 ^a^
**Distance to Regional Hospital** ^b^ **(kms)**	89 (32, 148)	73 (70, 85)
**Rurality** ^c^		
Rural	1 (25%)	4 (80%)
Small Rural	2 (50%)	1 (20%)
Remote	1 (25%)	0 (0%)
**Year PMH Model of Care Started**		
2016	1 (25%)	2 (40%)
2019	1 (25%)	0 (0%)
2020	2 (50%)	2 (40%)
2021	0 (0%)	1 (20%)
**Clinic Ownership**		
Public (Health Authority)	3 (75%)	0 (0%)
Private	1 (25%)	5 (100%)
**Physician Full Time Equivalent**		
Full time	1 (25%)	0 (0%)
No Physicians (Nurse Practitioner Only)	1 (25%)	0 (0%)
Part time	2 (50%)	5 (100%)
**Number of Physicians in Clinic**	2.00 (1.63)	5.60 (2.30)

^a^ n (%); Median (IQR)

^b^ Hospital services classified in BC using Level 1 (Community Health Centre) through Level 5 (Regional Hospital) of care description [27]

^c^ Based on geographic location, population, and type/amount of healthcare services available: Rural population 3500–20,000; Small Rural population 1000–3500; Remote population 0–1000 [27]

Compared to those that participated, the clinics that declined had a greater percentage of private clinic ownership, more clinic physicians, and more often provided care in larger rural communities that were somewhat closer to the regional hospital.

PHR adoption difficulties related to lack of systems interoperability (i.e., provider frustration with logging into two different systems and the manual effort of data entry between the EHR and PHR) and the COVID-19 pandemic (i.e., quick shift from in-person to virtual care, provider and patient priorities, and capacity to manage during unprecedented times). These issues also impacted the likelihood that PHR use would continue.

#### Implementation

Initially, the choice of the PHR vendor included the expectation of provincial interoperability standards to be in place. At the time of implementation, information exchange standards had not been confirmed in BC, and as such, EHR vendors prioritized their business strategies and chartered their own course leaving patient record systems and tools disparate and disconnected. To complicate implementation efforts, the COVID-19 pandemic deeply challenged the healthcare system and the capacity of providers, and care for patients shifted mostly to virtual care, making study recruitment and retention difficult.

With our commercial vendor, some system customizations were completed. One clinic requested a consent form for electronic communications. Another clinic requested a blood pressure log and photo submission form. Not all requests were able to be made (e.g., additional patient demographic fields) due to vendor capacity and priorities. Providers were offered a PHR refresher training in March 2020, but only one clinic accepted. Providers, with the help of clinic staff, managed initial patient access to the PHR. The study and access to the PHR ended in October 2020.

**Fig 2:**
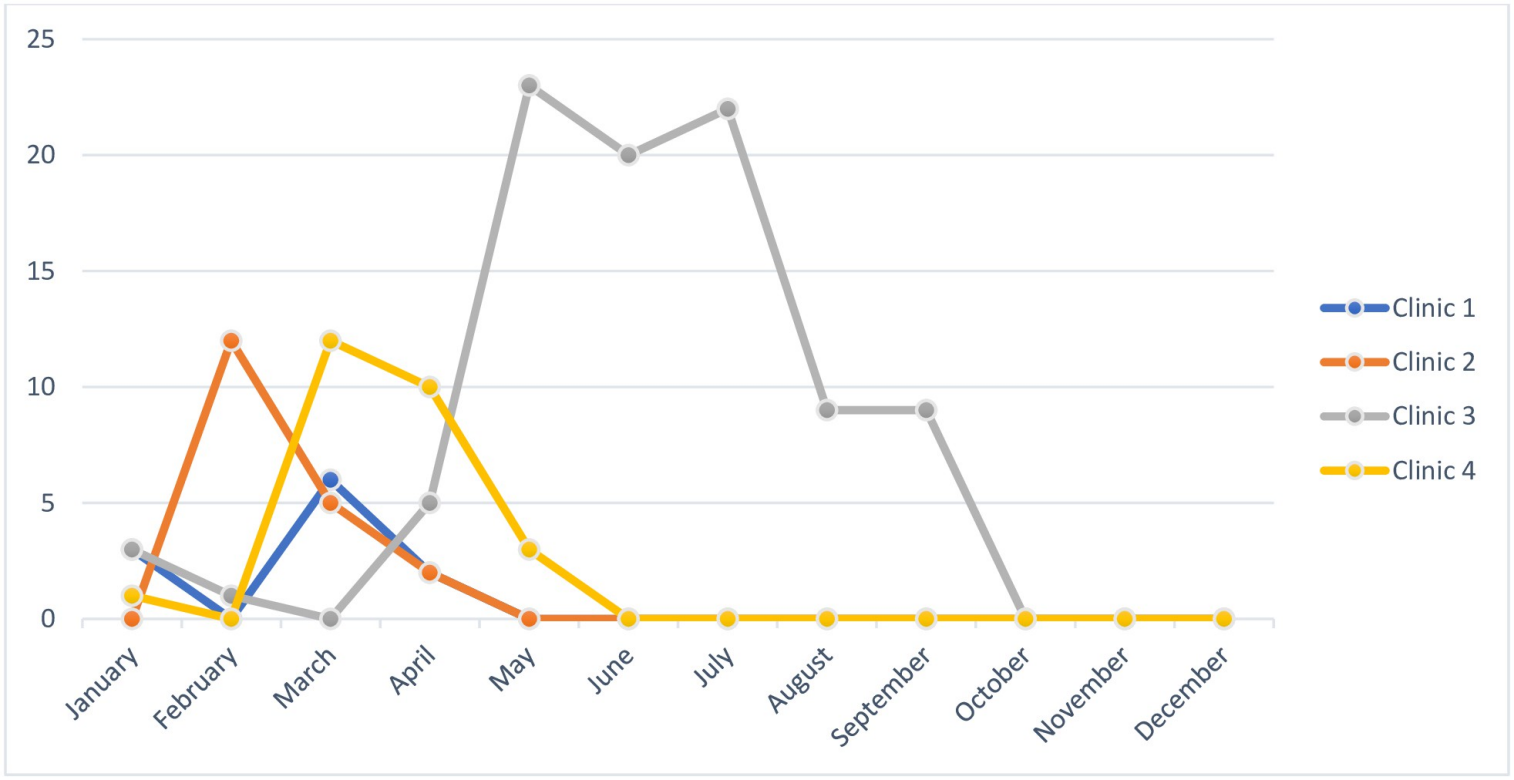
Number of Patients’ Initial Access to PHR by Month.

The number of patients given access by month is shown in Fig 2. The peak number of patients accessing the PHR occurred at the start of the study which also coincided with the early months of the pandemic. For one clinic, the use of photo submission during the shift to virtual care during the pandemic was identified as very valuable. The large spike in new implementations in May 2020 was a result of this clinic’s rapid uptake to support virtual care with a broader group of patients than the target population.

#### Maintenance

With the exception of one clinic, all clinics did not invite new patients to use the PHR after 5 months (Fig 2), with the providers citing barriers identified in Table 5. One clinic indicated that the benefit likely outweighed the barrier related to lack of PHR/EHR interoperability and would consider its continued use, if available, beyond the study.

## Discussion

In phase 1, clear distinctions emerged between patient and provider perspectives on types of data valued; agency, control, and boundaries; and potential negative patient impact. Patients emphasized health promotion data while providers focused on clinical data. In rural communities where there is a greater prevalence of unhealthy behaviors and chronic diseases compared to urban communities [28], both kinds of data are important to maximizing care, patient self-management, and health outcomes. Patient interest in using the PHR as a tool for health promotion may facilitate a paradigm shift, long needed in primary care, towards greater attention to illness prevention and healthy behaviors [29]. In a systematic review of patient and provider attitudes toward PHR use, the authors conclude that the provision of resources to understand and manage chronic conditions will promote adoption [30]. At present, having unique sections within a PHR for resources and storage and tracking of health behavior data without expectation of provider review might allow patient agency without overwhelming providers.

Aspects of PHR use that likely influence adoption were also identified in phase 1. Patients advocated for control of their complete health record, a finding consistent with other evidence indicating peoples’ beliefs they should control what information is shared and with whom [31]. Providers viewed the PHR as an opportunity to motivate and empower patients to participate in their care and decision making but recognized their role as gatekeepers of patient data. Along with access and engagement, control and contribution to health information and individual-level data have been identified as key barriers to person-enabled health systems [32].

Tethered and standalone PHRs are typically unable to provide a comprehensive health profile due to each containing different but relevant patient information. Interconnected PHRs capitalize on a collaborative, person-centered care model [33] and have great potential for rural contexts to bridge disparate patient and provider positions on patient-generated data usage and agency and control. In Ruhi and Chugh’s [33] high-level functional utility model of PHRs, using a consumer-value framework, content (e.g., medication history, patient diaries), connectivity (e.g., self-help dashboards, health reminders) and collaborative features (e.g., authorizations and permissions, online support groups) that bring value to patients must be considered. This will be critical to address in a future PHR implementation.

The reach of the PHR implementation was poor with only 16% of invited patients participating in PHR use, although an additional 127 used the PHR. This may, in part, be related to lack of provider endorsement which is a key component of patient PHR adoption. Beliefs by physicians that PHRs can improve patient relationships and quality of care is associated with patient’s willingness to use them [34]. Yousef et al. identified 8 key provider factors related to patient use of a PHR including showing the relevance of PHRs and increasing the perceived value by focusing on unique services in addition to providing education and training, integration into the existing technology, aligning PHR functions with workflow, offering incentives to individuals or teams, making information accessible, and supporting asynchronous and bidirectional communication [35]. In clinic 3 of our study, providers saw great value in the PHR functions during the pandemic, allowing for virtual visits and communication of patient-generated data resulting in many additional patients recruited to use of the PHR. This PHR use is in-line with a study which identified an active patient portal as a key variable for effective virtual visits during the pandemic [36].

Effectiveness appeared to be poor based on lack of positive impact on patients’ quality of life, self-efficacy, patient activation, and neutral satisfaction overall, but moderate with respect to use for collection and sharing of patient-generated data by patients and providers in the study. Barriers identified included lack of integration of patient and provider systems, no financial incentive, added workload, patient and provider expectations of use, and technical difficulties. It was difficult to separate the true impact of the PHR implementation on patient metrics from the impacts of COVID-19. It is possible that patient outcome scores might have gone down during the pandemic and that access to the PHR helped to sustain patient quality of life and self-efficacy. Several studies have demonstrated reduced patient quality of life in the aftermath of the pandemic [37–39]. In addition, half of patient participants reported poor physical health before and after the PHR implementation and additional time may have been needed to observe positive change for these patients.

The PHR adoption was moderate in our study, with 44% of invited clinics participating, including 71% of providers across those practices. There was a bias towards participation by smaller rural and remote practices. This finding is supported by an older U.S. national survey in which rural physicians and those with lower patient volumes were more willing to use PHRs [34].

Implementation was seriously handicapped by lack of anticipated provincial interoperability standards and system integration issues and the onset of the COVID-19 pandemic. In a review of PHR functionalities and implementation, Harahap et al. [40] identified interoperability, security and privacy, usability, data quality, and personalization as important factors, with lack of interoperability as a key implementation issue. Digital health transformation at the system-level is needed to realize PHR interoperability providing standard definitions for data exchange and cooperation with provider and organizational systems. Lack of integration between the EHR and PHR also contributed to poor PHR maintenance, with most practices discontinuing use of the PHR within 5 months. However, this timeframe did afford insights into the PHR features that patients and providers found most useful.

RE-AIM offers a comprehensive framework in which to guide a process evaluation of the PHR implementation and its impact in rural, team-based clinical practice. In a systematic review examining RE-AIM use over time, the authors indicate only a small percentage of articles reported on all 5 dimensions and criteria within a RE-AIM dimension and qualitative methods were rarely used [41]. In our study, all aspects of the framework were incorporated in the process evaluation including interviews to capture a rich set of data to optimally describe the PHR implementation. Highlighted as a challenge by Gaglio et al. [41] in their systematic review, a clear distinction between Reach and Adoption was established in our study but challenges were observed with the use of a valid denominator for Reach. The implementation strategy, using providers for recruitment of patients, left uncertain how many patients were eligible to participate.

As this was an exploratory study, care must be taken when transferring the findings. For example, the study represented variation in rural primary care practices but with small care teams and few patient participants, which may not be representative of larger rural practice settings. As the study was conducted during the pandemic, recruitment and retention of patients was particularly challenging.

## Conclusions

The comparison of patient and provider pre-implementation perspectives of PHRs in phase 1 offered some similarities but also differences. While both patients and providers agreed on PHR’s patient-oriented focus and information sharing benefits, they differed on preferred types of patient-reported data (health vs. wellness focus) and PHR control and communication boundaries. Understanding the views shared in this study lays groundwork for further research and can assist in optimizing the implementation and use of upcoming PHR systems.

The process evaluation of phase 2 identified benefits and obstacles with the implementation and use of a PHR in team-based primary care. The first steps for a successful integration of the PHR within today’s evolving EHR ecosystem and team-based care approach is to address barriers uncovered in this study; many of which can be addressed through a systems approach which focuses on EHR-PHR integration, patient-preferences, and provider training and support. As this was an exploratory study, further research is needed to inform priorities and approaches for future implementation success of a PHR in rural primary care.

## Supporting information

S1 Appendix Pre-Implementation Focus Group Guide. (DOCX)

S2 Appendix Description of Measures. (DOCX)

S3 Appendix Provider Post-Implementation Interview and Focus Group Guide. (DOCX)

S4 Appendix Pre-Implementation Patient and Provider Demographics. (DOCX)
