# Supplementary material for: Personal Health Record implementation in rural primary care: A descriptive exploratory study using RE-AIM framework
Source: PLOS Digit Health. 2024 Jun 26;3(6):e0000537. doi: 10.1371/journal.pdig.0000537 (PMC11207137; doi:10.1371/journal.pdig.0000537)
Supplement: S1 Appendix — (DOCX) [file pdig.0000537.s001.docx]

# S1 Appendix: Pre-Implementation Focus Group Guide

Facilitator Instructions:

Good morning/afternoon/evening. Thank you for taking the time to join us today. My name is __________ and I am a ___<role>____________ on this project. As indicated in the consent form, we want to understand your perceptions of personal health records in healthcare. We are conducting several focus groups with providers and patients in the Kootenay Boundary. Do you have any questions about the study or this session before we begin?

| Provider Questions | Tell us about your understanding of Personal Health Records? |
| --- | --- |
|  | What is your perception of the value of using PHRs in your work? |
|  | What types of communication methods do you think you would prefer to use with your patients? |
|  | Tell us about your interpretation of ‘patient-generated data’. How are these data differ from data gathered by the healthcare team, if at all? |
|  | What is most important to you about integration patient-generated data into your work? |
| Patient Questions | Tell us about your understanding of Personal Health Records? |
|  | What is your perception of the value of using PHRs in your daily life? |
|  | What types of communication methods do you think you would prefer to use with your healthcare provider team? |
|  | Tell us about your interpretation of ‘patient-generated data’. How are your data different from data gathered by your healthcare team, if at all? |
|  | What does it mean to you to share your personally generated data with your healthcare providers? |
|  | What is most important to you about sharing your personal data with the healthcare team? |
