# Supplementary material for: Personal Health Record implementation in rural primary care: A descriptive exploratory study using RE-AIM framework
Source: PLOS Digit Health. 2024 Jun 26;3(6):e0000537. doi: 10.1371/journal.pdig.0000537 (PMC11207137; doi:10.1371/journal.pdig.0000537)
Supplement: S2 Appendix — (DOCX) [file pdig.0000537.s002.docx]

# S2 Appendix: Description of Measures

| **Measure Name** | **Measure Description** | **Pre/Post** |
| --- | --- | --- |
| PHR familiarity and expectations | A 16 item questionnaire measuring familiarity with personal health records, technology, and expectations for PHR use and impact on health management. | Pre |
| PHR satisfaction | A 18 item questionnaire measuring satisfaction with PHR use | Post |
| Patient activation | A 12 item questionnaire Partners in Health Scale measuring patient activation and empowerment in self-management chronic health conditions. | Pre/post |
| Patient satisfaction | A 21 item questionnaire measuring patient satisfaction with primary health care services, adapted from CAHPS Clinician & Group Adult Survey | Pre/post |
| Quality of life | A 14 item questionnaire measuring health related quality of life, focusing on general health, activity limitations, and number of health days | Pre/post |
| Self-Efficacy | The Self-Efficacy for Managing Chronic Disease 6 item questionnaire measuring confidence chronic disease self-management | Pre/post |
