# Supplementary material for: Personal Health Record implementation in rural primary care: A descriptive exploratory study using RE-AIM framework
Source: PLOS Digit Health. 2024 Jun 26;3(6):e0000537. doi: 10.1371/journal.pdig.0000537 (PMC11207137; doi:10.1371/journal.pdig.0000537)
Supplement: S3 Appendix — (DOCX) [file pdig.0000537.s003.docx]

# S3 Appendix: Provider Post-Implementation Interview and Focus Group Guide

Facilitator Instructions:

1. Good morning/afternoon/evening. Thank you for taking the time to join us today. My name is __________ and I am a ___<role>____________ on this project.
2. Confirm identity (speaking to the right person, group)
3. Confirm this is still a good time to talk (need a total of about 60 minutes), if not reschedule
4. Re-affirm consent to participate
5. Remind them about the purpose of the call (This interview is to help us understand the meaning, value and use of patient-reported data for personal health records within rural, team-based primary care in BC)
6. Thank them for participating, acknowledge their opinions as valuable to us
7. Ask if they have any questions about the study or this session before we begin?

**Perceptions of Benefits/Outcomes**

1. What did you expect from the PHR before you started using it? Did the PHR meet your expectations? If so how? If not, why not?
2. What features of the PHR were the most useful/helpful?  Talk about a situation where you found it was useful/helpful. Talk about a situation where it wasn’t particularly helpful.
3. What role did the PHR play in your management and care of patients? Prompt: What did you find valuable as a physician using a PHR? What was not valuable about the PHR?
4. What were your biggest challenges in using the PHR?
5. What role do you think the PHR played in your patient’s health? Health management?
6. Now that you have had a chance to use the PHR what would you tell other physicians? Patients currently not using the PHR?
7. Now that you have had a chance to use the PHR, what, if anything, would you change about the PHR? What features would make it more useful for you and your patients?
