# Supplementary material for: Personal Health Record implementation in rural primary care: A descriptive exploratory study using RE-AIM framework
Source: PLOS Digit Health. 2024 Jun 26;3(6):e0000537. doi: 10.1371/journal.pdig.0000537 (PMC11207137; doi:10.1371/journal.pdig.0000537)
Supplement: S4 Appendix — (DOCX) [file pdig.0000537.s004.docx]

# S4 Appendix: Pre-Implementation Patient and Provider Demographics

## Pre-Implementation Summary of Patient Demographics

| **Variable** | **n** | **Mean** | **SD** | **Range** |
| --- | --- | --- | --- | --- |
| **Age** | 9 | 59.4 | 17.69 | 28-82 |
| **Distance From Clinic** (Kms) | 9 | 5.8 | 7.48 | 1-25 |
|  | | | | |
|  |  |  | **Frequency** | **%** |
| **Sex** |  |  |  |  |
| female |  |  | 8 | 88.9 |
| male |  |  | 1 | 11.1 |
| **Marital Status** |  |  |  |  |
| Divorced |  |  | 2 | 22.2 |
| Married |  |  | 3 | 33.3 |
| Single |  |  | 2 | 22.2 |
| Widowed |  |  | 2 | 22.2 |
| **Ethnicity** |  |  |  |  |
| Aboriginal |  |  | 2 | 22.2 |
| Caucasian |  |  | 6 | 66.7 |
| Metis |  |  | 1 | 11.1 |
| **Education** |  |  |  |  |
| College |  |  | 6 | 66.7 |
| Some college |  |  | 2 | 22.2 |
| High school |  |  | 1 | 11.1 |
| **Income** |  |  |  |  |
| <25,000 |  |  | 3 | 33.3 |
| 25,000-50,000 |  |  | 2 | 22.2 |
| 51,000-75,000 |  |  | 2 | 22.2 |
| >75,000 |  |  | 2 | 22.2 |
| **Medication**  Number of different prescription medications |  |  |  |  |
| None |  |  | 3 | 33.3 |
| 1-2 |  |  | 3 | 33.3 |
| 3-5 |  |  | 3 | 33.3 |
| **Overall Health** |  |  |  |  |
| Excellent |  |  | 3 | 33.3 |
| Good |  |  | 3 | 33.3 |
| Fair |  |  | 3 | 33.3 |
| **Mental Health** |  |  |  |  |
| Excellent |  |  | 5 | 55.6 |
| Good |  |  | 1 | 11.1 |
| Fair |  |  | 3 | 33.3 |
| **Technology**  use of information and communications technologies |  |  |  |  |
| Always use |  |  | 5 | 55.6 |
| Regular use |  |  | 4 | 44.4 |

## Pre-Implementation Summary of Provider Demographics

| **Variable** | **n** | **Mean** | **SD** | **Range** |
| --- | --- | --- | --- | --- |
| **Practice Length** (years) | 16 | 12.7 | 5.47 | 1-38 |
| **EHR:** length time working with EHR (years) | 17 | 8.6 | 4.68 | 1-20 |
|  | | | | |
|  |  |  | **Frequency** | **%** |
| **Sex** |  |  |  |  |
| female |  |  | 14 | 82.4 |
| male |  |  | 3 | 17.6 |
| **Professional Category** |  |  |  |  |
| physician |  |  | 8 | 47.1 |
| medical office assistant |  |  | 5 | 29.4 |
| nurse practitioner |  |  | 2 | 11.8 |
| registered nurse |  |  | 2 | 11.8 |
| **Participating Clinic** |  |  |  |  |
| Clinic I |  |  | 5 | 29.4 |
| Clinic II |  |  | 4 | 23.5 |
| Clinic III |  |  | 4 | 23.5 |
| Clinic IV |  |  | 3 | 17.6 |
| Clinic V |  |  | 1 | 5.9 |
| **Technology User Type**  type of user of information and communications technologies |  |  |  |  |
| Advanced |  |  | 6 | 35.4 |
| Average |  |  | 9 | 52.9 |
| Basic |  |  | 2 | 11.8 |
